# Spectral probabilities of top-down tandem mass spectra

**DOI:** 10.1186/1471-2164-15-S1-S9

**Published:** 2014-01-24

**Authors:** Xiaowen Liu, Matthew W Segar, Shuai Cheng Li, Sangtae Kim

**Affiliations:** Department of BioHealth Informatics, Indiana University-Purdue University Indianapolis, 535 W. Michigan Street, 46202 Indianapolis, IN USA; Center for Computational Biology and Bioinformatics, Indiana University School of Medicine, 410 West 10th Street, HS 5000, 46202 Indianapolis, IN USA; Department of Computer Science, City University of Hong Kong, 83 Tat Chee Avenue, Kowloon, Hong Kong SAR, China; Biological Sciences Division, Pacific Northwest National Laboratory, 902 Battelle Boulevard, 99352 Richland, WA USA

**Keywords:** mass spectrometry, spectral probabilities, dynamic programming

## Abstract

**Background:**

In mass spectrometry-based proteomics, the statistical significance of a peptide-spectrum or protein-spectrum match is an important indicator of the correctness of the peptide or protein identification. In bottom-up mass spectrometry, probabilistic models, such as the generating function method, have been successfully applied to compute the statistical significance of peptide-spectrum matches for short peptides containing no post-translational modifications. As top-down mass spectrometry, which often identifies intact proteins with post-translational modifications, becomes available in many laboratories, the estimation of statistical significance of top-down protein identification results has come into great demand.

**Results:**

In this paper, we study an extended generating function method for accurately computing the statistical significance of protein-spectrum matches with post-translational modifications. Experiments show that the extended generating function method achieves high accuracy in computing spectral probabilities and false discovery rates.

**Conclusions:**

The extended generating function method is a non-trivial extension of the generating function method for bottom-up mass spectrometry. It can be used to choose the correct protein-spectrum match from several candidate protein-spectrum matches for a spectrum, as well as separate correct protein-spectrum matches from incorrect ones identified from a large number of tandem mass spectra.

## Background

Peptide and protein identification in mass spectrometry (MS)-based proteomics involves searching tandem mass spectrometry (MS/MS) spectra against a protein database using a search engine. In bottom-up MS, most search engines calculate a similarity score between a spectrum and a peptide and report a best-scoring peptide-spectrum match (PSM) for each spectrum [1–5]. A PSM is *correct* if the spectrum is generated from the matched peptide. It is vital to distinguish correct PSMs from those incorrect ones.

Two main approaches have been proposed to address this problem. In the first approach, a large data set of MS/MS spectra is searched against a concatenated target-decoy protein database to find a best-scoring PSM for each spectrum, and the PSM is reported if its score exceeds a prespecified threshold. The false discovery rate (FDR) of the reported PSMs is estimated based on the fact that the number of decoy hits and the number of incorrect target hits are approximately the same [6]. This approach is simple and powerful when a large population of PSMs is reported. However, it fails to decide the correctness of single PSMs. In addition, it is unable to compute accurate FDRs when the target protein database is small (e.g., a database with only one protein) or when only a small number of PSMs are reported [7].

In the second approach, the statistical significance (*E*-value or *p*-value) of a PSM is computed for determining the correctness of the PSM. Due to the complexity of MS/MS spectra, many statistical models have limited accuracy. By contrast, Kim *et al*. proposed a probabilistic method for computing *spectral probabilities* and statistical significance of PSMs [8]. This method achieves high accuracy, but it is not obvious how to extend it to PSMs with post-translational modifications (PTMs).

With the rapid developments in instrumentation, top-down MS, which analyzes intact proteins or long peptides, has become available in many laboratories. More than a thousand proteins can be identified in a single top-down MS experiment [9] and many methods have been proposed for identification of proteoforms using top-down tandem mass spectra [10–17]. Although the evaluation of PSMs in bottom-up MS has been intensively studied, no systematic studies have been carried out for evaluating protein-spectrum matches (PrSMs) in top-down MS. Similar to bottom-up MS, there is now an increasing demand to accurately estimate the statistical significance of *single* PrSMs. For instance, a top-down MS/MS spectrum can be matched to two different proteins: one contains a PTM; the other does not. Comparing the *E*-values of the two PrSMs can determine which one is better. Meng *et al*. developed a Poisson model for the problem, but the model does not include PTMs [18]. As top-down MS/MS spectra are often mapped to proteoforms with PTMs, accurate estimation of statistical significance of PrSMs with PTMs is useful and challenging. We proposed a method for *E*-value computation of PrSMs by breaking a protein into several sub-proteins without PTMs, but it is extremely time consuming [17]. In this paper, we study an extended generating function method for accurately computing *spectral probabilities* and statistical significance of PrSMs in top-down MS. Our method naturally extends the generating function method in bottom-up MS [8]. Spectral probabilities reported by the extended generating function method are further utilized for estimating FDRs of identified PrSMs using a method proposed in [7], in which decoy databases are not needed. Experiments show that the extended generating function method achieves high accuracy in computing spectral probabilities and FDRs.

## Methods

A top-down MS/MS spectrum generated from a protein consists of a precursor mass, corresponding to the molecular mass of the protein, and a list of peaks, corresponding to fragment ions of the protein. Each peak represents the mass-to-charge ratio and the abundance of the fragment ion. The *residue mass* of a spectrum *S* is defined as M(*S*) = *PrecursorMass − WaterMass*, where *PrecursorMass* is the precursor mass of the spectrum, and *WaterMass* is the mass of a water molecule. Because top-down MS/MS spectra are very complex, and the charge states of most fragment ions are high, high mass resolution and high mass accuracy spectra are absolutely required. The first step in top-down spectral interpretation is usually spectral deconvolution, which converts a complex top-down spectrum to a list of monoisotopic neutral masses (a deconvoluted spectrum) [19, 20]. The neutral masses are further converted to a list of *prefix residue masses (PRMs)* corresponding to the masses of protein prefixes [21]. For a collision-induced dissociation (CID) spectrum, the PRM spectrum is generated as follows: (1) the residue mass of the experimental spectrum is added to the PRM spectrum; (2) for each neutral mass *x* extracted from the experimental spectrum, two masses *x* and *PrecursorMass − ×* are added to the PRM spectrum. If mass *x* corresponds to a protein suffix (prefix), then mass *PrecursorMass − ×* corresponds to a protein prefix (suffix) [22]. The proposed extended generating function method can be applied to all types of spectra, such as CID and electron-transfer dissociation (ETD) spectra, because all these types of spectra can be converted to PRM spectra. All masses in PRM spectra are discretized by scaling the masses with a constant and rounding the values to integers [23]. For highly accurate top-down spectra, a scaling constant 274.335215 is used (e.g. mass(*G*) = 57.021464 × 274.335215 = 15642.995586 *≈* 15643) to reduce the rounding error to 2.5 parts per million (ppm) [22]. In the following analysis, we assume that only PRM spectra with integer masses are studied and peak intensities are ignored.

### Scores of PrSMs

A PRM spectrum *S* is represented as an ordered list of integer masses, in which the largest one is M(*S*). Let  be the set of the 20 standard amino acids with integer residue masses M(*r*) for  (the residue masses of amino acids are discretized using the same discretization method for PRM spectra). The residue mass of *r* is also denoted as *‖r‖*. The *residue mass* M(*P*) of a protein *P* is the sum of the residue masses of all amino acids of the protein. It differs from the molecular mass of the protein by the mass of a water molecule. A protein *P* with *m* amino acids is associated with an ordered list of integer masses *p*_1_*< p*_2_*<*. . . *< p*_*m*_, where *p*_*i*_ is the sum of the residue masses of the first *i* amino acids and *p*_*m*_ = M(*P*).

If the residue masses of spectrum *S* and protein *P* are the same value *N* , the *mass count score* of *S* and *P* is the number of shared masses (except for the residue mass *N*) in *S* and *P*, denoted by CScore(*S*, *P*). The *mass shift* of a PTM is the mass difference between the modified form (with the PTM) and the unmodified form of an amino acid residue. When a PTM occurs at the *i*th amino acid of *P* and the mass shift *d* of the PTM is positive, the modified form of *P* is denoted by *Q*_*i*__,__*d*_(*P*) = {*p*_1_, *p*_2_, . . ., *p*_*i−*__1_, *p*_*i*_ + *d*, . . ., *p*_*m*_ + *d*}. When the mass shift of the PTM is a negative value *−d*, *Q*_*i*__,__*−d*_(*P*) = {*p*_1_, *p*_2_, . . ., *p*_*i−*__1_, *p*_*i*_*− d*, . . ., *p*_*m*_*− d*}. In addition, if a mass in *p*_*i*_*− d*, . . ., *p*_*m*_*− d* is negative or not greater than *p*_*i−*__1_, the mass is removed from *Q*_*i*__,__*−d*_(*P*). Let  be the set of all modified proteins of *P* with a PTM of mass shift *d*. When the protein is not ambiguous, we use shortened notations . To identify an experimental PRM spectrum *S* generated from protein *P* with a PTM, one can find the mass shift *d* of the PTM by comparing the residue masses of *S* and *P* , and compute the mass count score between *S* and each of the modified proteins in  to find the best match. The *PTM mass count score* of *S* and *P* is defined as  CScore(*S*, *Q*), where *d* = M(*S*) *−* M(*P*).

### Random proteins

Let Pr(*r*) be the probability that an amino acid  is observed at a position in a random protein. In practice, the frequencies of amino acids in the Swiss-Prot database [24] can be used to estimate Pr(*r*). The probability that a random protein *P* with amino acids *r*_1_*r*_2_ . . . *r*_*m*_ is observed is

where *L* represents the length of the random protein. To simplify computation, a uniform probability Pr(*L* = *m*) = 1*/MaxLength* is chosen, where *MaxLength* is the length of the longest protein in the Swiss-Prot database. Despite the difference between the uniform distribution and the distribution of protein length in the target protein database, experimental results showed the uniform distribution does not introduce large errors into the computation of spectral probabilities.

### Spectral probabilities

Let  be the set of negative/positive mass shifts of allowed PTMs. Any number in  is a valid mass shift. Let *S* be an experimental PRM spectrum and *P* a random protein. The residue mass difference between *S* and *P* is a random variable *D* = M(*S*) *−* M(*P* ). The *spectral probability* of *S* with respect to a threshold *t* and one PTM is the probability that the residue mass difference  and PScore(*S*, *P* ) *≥ t*:1

where 1 in SpecProb(*S*, *t*, 1) represents one PTM. From the definition of PScore(*S*, *P*),2

Computing SpecProb(*S*, *t*, 1) accurately and efficiently is a problem that has not been solved. In the following subsections, we propose two upper bounds of SpecProb(*S*, *t*, 1). The two upper bounds can be calculated accurately and efficiently using dynamic programming algorithms. The second upper bound is better than the first one and is used for estimating SpecProb(*S*, *t*, 1). Since the second upper bound is larger than SpecProb(*S*, *t*, 1), a constant *K* is introduced for correcting errors in estimated spectral probabilities. In practice, the value of *K* can be estimated from training data sets.

### The first upper bound of spectral probabilities

Based on Equation (2) and the union bound of probabilities,3

Let *q* denote the right hand part of the above inequality. The value of *q* is an upper bound of SpecProb(*S*, *t*, 1). Next, we describe a dynamic programming algorithm for computing the value of *q*. The algorithm is an extension of the generating function method in [8]. In this algorithm, a spectrum *S* with a residue mass *N* is represented as a 0/1 vector *S* = *s*_1_*s*_2_ . . . *s*_*N*_, where *s*_*i*_ = 1 if the spectrum has a prefix residue mass *i* and 0 otherwise. For example, a spectrum with a PRM list {2, 5, 8, 10} (10 is the residue mass of the spectrum) is represented as 0100100100. We first study the case where all mass shifts are positive; negative mass shifts will be discussed at the end of this subsection. A three dimensional table *T* (*i*, *j*, *k*) is computed to acquire the upper bound, where *i* is the number of PTMs in modified proteins. Let *S*[1 : *j*] be the subspectrum *s*_1_*s*_2_ . . . *s*_*j*_ of *S*. The residue mass of *S*[1 : *j*] is *j*. The value *T* (0, *j*, *k*) is the probability that M(*P*) = *j* and the mass count score CScore(*S*[1 : *j*], *P*) = *k*. Let  be set of all proteins with a residue mass *j*. We define a function: *f*(*S*, *P*, *k*) = 1 if CScore(*S*, *P*) = *k*; 0 otherwise. Then,4

Suppose *P* contains *m* amino acids and the residue mass of *P* is *j*. If the last amino acid of *P* is *r*, then *j − ‖r‖* is the prefix residue mass of the first *m −* 1 amino acids of *P*, where *‖r‖* is the residue mass of *r*. In the vector representation of *S*, if *S* contains a prefix residue mass *j − ‖r‖*, *s*_*j−‖r‖*_ = 1; otherwise, *s*_*j−‖r‖*_ = 0. The recurrence function for computing *T*(0, *j*, *k*) was given in [8]:5

Let *D*_*j*_ = *j −* M(*P* ), the random variable representing the difference between *j* and the residue mass of random protein *P*. The value *T*(1, *j*, *k*) is the sum of probabilities6

Suppose the residue mass of protein *P* is *j − d*, that is, . Let *m* be the number of amino acids in *P* and *Q*_*m*__,__*d*_ the modified protein of *P* whose PTM is on the last amino acid. Because the first *m−*1 masses of *Q*_*m*__,__*d*_ are unchanged compared with *P*,

Combined with Equation (4),7

Let *r* be the last amino acid of *P* and *P*' the protein containing the first *m −* 1 amino acids of *P*. All the *m −* 1 masses in *Q*_*l*__,__*d*_(*P*'), 1 *≤ l ≤ m −* 1, are the same to the first *m −* 1 masses in *Q*_*l*__,__*d*_(*P*). While the *m −* 1th mass *j − ‖r‖* in *Q*_*l*__,__*d*_(*P*) is included in the computation of mass count scores, the mass *j − ‖r‖* in *Q*_*l*__,__*d*_(*P*') is not included because it is the residue mass. Thus,

It follows8

Combining the fact that *Pr*(*P*) = *Pr*(*P*')*Pr*(*r*) and Equations (6) and (8),9

With Equations (6), (7) and (9), the recurrence function for *T*(1, *j*, *k*) is10

When PTMs with negative mass shifts *d* are allowed, *j − d* in Equation (10) is larger than *j*. The value *T*(1, *j − d*, *k*) has not been computed when *T*(1, *j*, *k*) is computed, making Equation (10) invalid. To avoid this problem, a short amino acid sequence *g* is introduced to guarantee that *j − d −* M(*g*) *< j*. Let  be the set of all amino acid sequences *g* = *r*_1_*r*_2_ . . . *r*_*l*_ satisfying M(*g*) *> −d* and M(*r*_1_*r*_2_ . . . *r*_*l−*__1_) *≤ −d* (*d* is negative). Equation (10) is modified to11

where *‖g‖* is the residue mass of *g*, and  for a sequence *g* = *r*_1_*r*_2_ . . . *r*_*l*_. The value of *q* is , where *N* and *n* are the residue mass and the number of masses of *S*, respectively. The time complexity for computing *T*(0, *j*, *k*) and *T*(1, *j*, *k*) is *O*(*N · t · z*), where *z* is the sum of the sizes of  and all , .

### The second upper bound of spectral probabilities

The only difference between two modified proteins *Q*_*i*__,__*d*_ and *Q*_*i*__+1,__*d*_ is the *i*th mass. If *p*_*i*_ in *P* (which is not changed in *Q*_*i*__+1,__*d*_) does not equal any mass in *S*, then CScore(*S*, *Q*_*i*__+1,__*d*_) *≤* CScore(*S*, *Q*_*i*_,_*d*_). Based on this observation, if *p*_*i*_ does not equal any mass in *S*, *Q*_*i*__+1,__*d*_ is removed from . In this way, a new set  is acquired containing *Q*_1,*d*_and all *Q*_*i*_,_*d*_ satisfying that *p*_*i−*__1_ equals a mass in *S*. It follows that . From Equation (1) and the union bound of probabilities,

Let *q*' denote the right hand part of the above inequality. Compared with *q*, the value of *q*' is a better upper bound for SpecProb(*S*, *t*, 1). Similar to the method for computing *q*, we fill out a three dimensional array *T*(*i*, *j*, *k*) for computing *q*'. The recurrence function for filling out *T*(0, *j*, *k*) is the same to Equation (5). We change the definition of *T*(1, *j*, *k*) by replacing  with  in Equation (6). To compute *T*(1, *j*, *k*), the second and third terms of the right-hand part of Equation (11) should be changed so that only the probabilities for the modified proteins in  are summed up.

Similar to the proof of Equation (7), consider a random protein . Let *Q*_*m*_,_*d*_ be the modified protein of *P* whose PTM is on the last amino acid, and *r* the last amino acid of *P*. If , then *j − d − ‖r‖* is a mass in *S* or *j − d − ‖r‖* = 0 (in the extreme case that *P* contains only one amino acid, *j − d − ‖r‖* = 0), and vice versa. Therefore, if *j − d − ‖r‖* = 0 or *s*_*j−d−‖r‖*_ = 1, then Pr(*P* ) *· f*(*S*[1 : *j*], *Q*_*m*_,_*d*_, *k*) is included in the computation of *T*(1, *j*, *k*).

For a positive mass shift *d*, we define  as the set of amino acids *r* ∈ *R* satisfying that *j − d − ‖r‖* = 0 or the element *s*_*j−d−‖r‖*_ = 1. For a negative mass shift *d*, we introduce a set  of amino acid sequences *g* = *r*_1_*r*_2_ . . . *r*_*l*_ satisfying: (1) M(*g*) *> −d*, (2) M(*r*_1_*r*_2_ . . . *r*_*l−*__1_) *≤ −d*, and (3) *j − d − ‖g‖* = 0 or the element *s*_*j−d−‖g‖*_ = 1. Then Equation (11) is changed to:12

and . The time complexity for computing *T*(0, *j*, *k*) and *T*(1, *j*, *k*) is similar to the method in the previous subsection.

Since the scores CScore(*S*, *Q*) for  are not independent, *q*' is usually larger than the spectral probability SpecProb(*S*, *t*, 1). To estimate SpecProb(*S*, *t*, 1) more accurately, *q*' is multiplied by a constant value *K* for correction:13

### *P*-values and *E*-values

Let , where *N* is the residue mass of *S*. From table *T*(0, *j*, *k*) described in the previous subsection, we can compute the probability that the residue mass difference *D* between *S* and *P* is in :14

Using Equations (13) and (14), the *conditional spectral probability* of *S* with respect to threshold *t* and one PTM is15

Intact proteins may have N or C-terminal truncations, e.g., the removal of a signal peptide. If a top-down MS/MS spectrum is matched to an intact protein without N- or C-terminal truncations, the PrSM is called a *complete* PrSM. A PrSM matched to an intact protein with an N-/C-terminal truncation is called a *suffix*/*prefix* PrSM. An *internal* PrSM corresponds to an intact protein with both N- and C-terminal truncations.

Similar to the *E*-values defined in BLAST [25], the *E*-value of a PrSM describes the number of hits one can "expect" to see by chance when searching the spectrum against a protein database of a particular size. Suppose a complete PrSM contains one mass shift (PTM) in  and its PTM mass count score is *t*. We count the number *Z* of proteins in the target database with a residue mass in . The *E*-value of the complete PrSM is estimated as *Z ·* CSP(*S*, *t*, 1). The *p*-value of the PrSM is estimated as 1 *−* (1 *−* CSP(*S*, *t*, 1))^*Z*^.

For prefix, suffix and internal PrSMs, we count the numbers *Z*_*p*_, *Z*_*s*_, and *Z*_*i*_ of prefixes/ suffixes/internal sub-proteins in the target database with a residue mass in . Because some prefixes/suffixes/internal sub-proteins are not independent, a constant factor *C*_*p*_/*C*_*s*_/*C*_*i*_ is multiplied in the computation of *E*-values of prefix/suffix/internal PrSMs for correction. For example, if a prefix PrSM contains one mass shift (PTM) in  and its PTM mass count score is *t*, the *E*-value of the PrSM is estimated as *C*_*p*_*· Z*_*p*_*·* CSP(*S*, *t*, 1).

### Multiple PTMs

The dynamic programming algorithm for computing the second upper bound can be extended to estimate *E*-values of PrSMs with multiple PTMs. When multiple PTMs are allowed, we replace *T*(0, *j*, *k*) and *T*(1, *j*, *k*) in Equation (12) by *T*(*i*, *j*, *k*) and *T*(*i −* 1, *j*, *k*) to estimate spectral probabilities with respect to *i* PTMs:16

## Results

The extended generating function method, TD-GF (Top-Down Generating Function), was implemented in JAVA and tested on a desktop with a 3.3GHz (AMD Opteron 6204) CPU and 16 GB memory.

### Data sets

A *Salmonella typhimurium* (ST) data set [13] was used to test TD-GF. A protein mixture of ST was analyzed using an LTQ-Orbitrap (Thermo Fisher Scientific). MS and MS/MS spectra were collected at a resolution of 60,000 and 30,000, respectively. The experiment was repeated using gas-phase fractionation. A total of 14,041 collision-induced dissociation (CID) MS/MS spectra were acquired. The detailed experiment procedure can be found in [13].

The performance of TD-GF on proteoform identification was tested on an *Escherichia coli* (EC) data set. An EC cell lysate was separated by an intact protein reversed phase liquid-chromatography (RPLC) system and analyzed by an LTQ-Orbitrap Velos (Thermo Fisher Scientific). MS and MS/MS spectra was collected at a resolution of 60,000. A total of 3,704 higher-energy C-trap dissociation (HCD) MS/MS spectra were obtained.

### Spectral probabilities for PrSMs with one PTM

The accuracy of TD-GF was evaluated using two approaches based on conditional spectral probabilities (defined in Equation (15)) and FDRs.

#### Evaluation based on conditional spectral probabilities

To evaluate TD-GF, we generated a set of PrSMs with "correct" conditional spectral probabilities and compared the "correct" conditional spectral probabilities with those reported by TD-GF.

*Selection of PrSMs* Previous analysis results in [17] showed that most PrSMs identified in the ST data set contained no PTMs. To increase the number of identified PrSMs with one PTM, a mutated ST protein database was generated by adding a glycine residue to the middle of each protein sequence in the ST proteome. When the mutated ST protein database is used, a PrSM without PTMs can be identified as a PrSM with one PTM.

All MS/MS spectra in the ST data set were deconvoluted using MS-Deconv [20] and searched against the mutated ST proteome using MS-Align+ [17]. The error tolerances for precursor masses and fragment masses were set to 15 ppm, and carbamidomethylation was set as the fixed PTM. By restricting the search space to only complete PrSMs with one PTM, MS-Align+ identified 4,291 PrSMs. For each of 4,291 PrSMs, TD-GF was employed to compute the conditional spectral probability, which was used only for selecting PrSMs, not for evaluating TD-GF. The parameter *K* in Equation (13) was set to 1. Since blind PTM search was used in MS-Align+, the allowed mass shifts were set to  and , where *α* is the mass of a tryptophan (W) residue. The running time for computing conditional spectral probabilities was 726 minutes (about 12 hours). For 203 of the 4,291 complete PrSMs, the conditional spectral probabilities reported by TD-GF were in [10^*−5*^, 10^*−4*^]. The 203 PrSMs were selected for computing "correct" conditional spectral probabilities.

*Computation of "correct" conditional spectral probabilities* For each of the 203 PrSMs (spectra), a random database of 10^6^ proteins was generated. In the random database, the residue masses of all proteins are in , where *N* is the residue mass of the spectrum. The PTM mass count score between the spectrum and each protein in the database was computed; and the number *x* of proteins satisfying that the PTM mass count score *≥ t* was counted. The conditional spectral probability of the PrSM with respect to one PTM and threshold *t* was estimated as *x/*10^6^. Since the above method follows the definition of conditional spectral probabilities, the results are treated as "correct" conditional spectral probabilities. Finally, one PrSM was removed from the list of 203 PrSMs because the estimated conditional spectral probability (using a random database) was 0.

**Figure 1:**
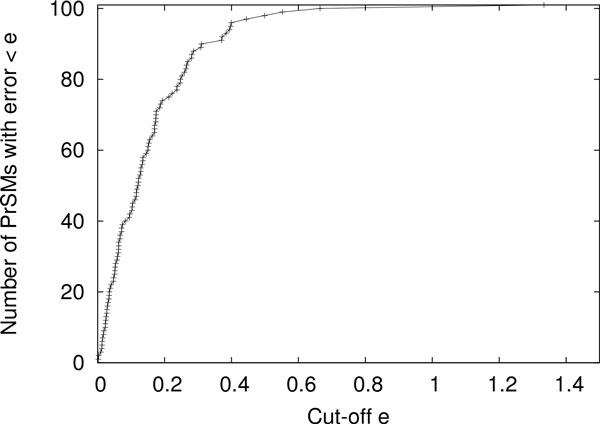
**A comparison of the conditional spectral probabilities (for PrSMs with one PTM) estimated by the random database-based method and TD-GF**. For each of the 101 test PrSMs, the error of the conditional spectral probability reported by TD-GF is computed. For each cut-off of *e*, the number of PrSMs with an error *< e* is counted.

*Evaluation of TD-GF* The 202 PrSMs were randomly divided into a training data set (101 PrSMs) and a test data set (101 PrSMs). The training data set was used to estimated the value of *K* in Equation (13). We set *K* = 1 (the value of *K* will be determined later) and used TD-GF to compute the conditional spectral probabilities for the training PrSMs. Let *p*_1_ and *p*_2_ be the conditional spectral probabilities of a PrSM estimated by the random database-based method and TD-GF, respectively. The error of *p*_2_ is defined as *e* = *|* log(*p*_1_) *−* log(*p*_2_)*|* (base 10). To minimize the average error of the conditional spectral probabilities reported by TD-GF, the best value of log(*K*) is the average of the log ratio . Using the training data set, *K* was set to the best value 0.55. In practice, the default values of *K* are learned from various types of training data, such as CID and ETD data, and are provided so that the users do not need to estimate *K* for their own data sets. With *K* = 0.55, TD-GF was employed to compute the conditional spectral probabilities for the test PrSMs. The errors of these conditional spectral probabilities were obtained by comparing them with the "correct" ones (Figure 1). The errors for 98 test PrSMs (97%) were *≤* 0.5. When the error is 0.5, there is about a three fold difference between the conditional spectral probabilities reported by the two methods. The results show that the spectral probabilities estimated by TD-GF are accurate for most of the test PrSMs.

#### Evaluation based on FDRs

With the spectral probabilities reported by TD-GF, the "estimated" FDR of a set of identified PrSMs for a cut-off *p*-value can be computed using the functions in [7]. For the same cut-off *p*-value, the "correct" FDR can be obtained by the target-decoy approach. Because the "estimated" FDR is based on the spectral probabilities reported by TD-GF, if the "estimated" FDR is similar to the "correct" FDR, then the spectral probabilities reported by TD-GF are accurate.

**Figure 2:**
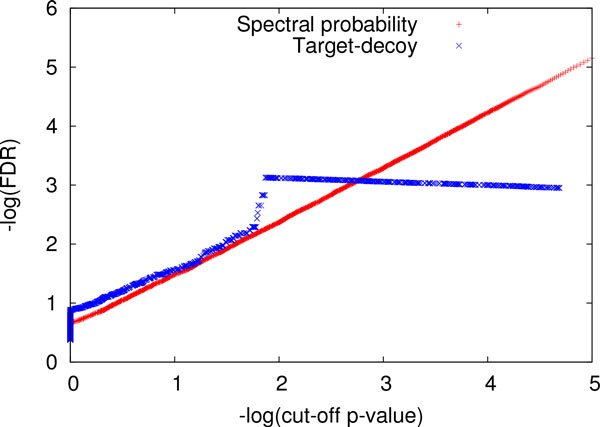
**A comparison of the FDRs of PrSMs with one PTM estimated by the target-decoy approach and computed based on spectral probabilities**. For a given cut-off *p*-value, the two reported FDRs are compared, and *−* log(FDR) (base 10) is plotted against *−* log(cut-off *p*-value) (base 10).

Using all the 4,291 complete PrSMs with one PTM, we computed "estimated" FDRs for cut-off *p*-values in {0.0001, 0.0002, . . ., 1.0000} based on spectral probabilities. In the target-decoy approach, all spectra were searched against a concatenated target and shuffled decoy protein database. Because the FDR reported by the target-decoy approach was 0 when the cut-off *p*-value was smaller than 8.27 × 10^*−4*^, we compared only the FDRs for cut-off *p*-values greater than 8.27 × 10^*−4*^ (Figure 2). The FDRs estimated based spectral probabilities were consistent with those reported by the target-decoy approach. For example, the target-decoy approach and the spectral probability approach reported cut-off *p*-values 0.0327 and 0.0262 for 1% FDR, respectively. The difference between the two *p*-values is only 0.0065, which is evidence that the spectral probabilities reported by TD-GF are accurate.

### Prefix, suffix and internal PrSMs

In this subsection, we describe the methods for estimating parameters *C*_*p*_, *C*_*s*_ and *C*_*i*_ introduced in Section Methods. A substring *a*_*i*_*a*_*i*__+1_ . . . *a*_*j*_ of a string *S* = *a*_1_*a*_2_ . . . *a*_*n*_ is denoted by *S*[*i* : *j*]. To estimate the parameter *C*_*p*_ for prefix PrSMs, a new random protein database was generated for each of the selected 202 PrSMs: (1) a total of 1,000 long random protein sequences with 1,200 amino acids each were generated, and (2) prefixes *S*[1 : 201], . . ., *S*[1 : 1200] were extracted from each of the 1,000 long protein sequences. In total, 10^6^ prefixes were added to the random protein database. The conditional spectral probabilities estimated using the new random databases are different from those using the random databases in Subsection "Computation of correct conditional spectral probabilities" because the protein sequences in the new random databases are not independent. Parameter *C*_*p*_ was estimated as the average ratio 0.693 between the probabilities computed based on the new databases and the random databases in Subsection "Computation of correct conditional spectral probabilities" for the 202 PrSMs. Parameter *C*_*s*_ can be set to the same to *C*_*p*_.

To estimated the parameter *C*_*i*_ for internal PrSMs, a third type of random protein databases were used: (1) a total of 4 long random protein sequences with 1200 amino acids each were generated, and (2) 2.5 × 10^5^ substrings *S*[*i* : *j*] (1 *≤ i ≤* 500, *i*+201 *≤ j ≤ i* + 700) of the each long protein sequence were added to the random database. Using the same method for computing *C*_*p*_, parameter *C*_*i*_ was estimated as 0.508.

### Spectral probabilities for PrSMs with two PTMs

**Figure 3:**
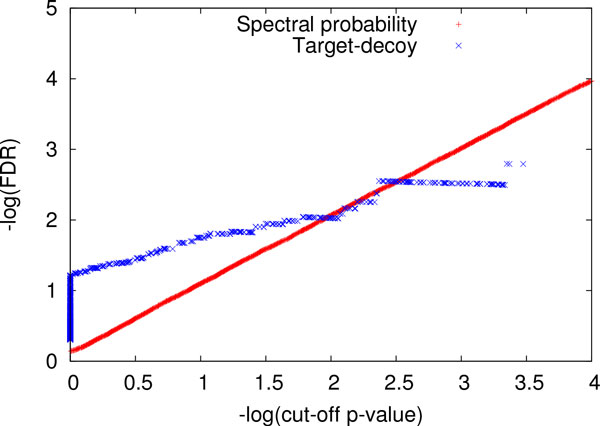
**A comparison of the FDRs of PrSMs with two PTMs estimated by the target-decoy approach and computed based on spectral probabilities**. For a given cut-off *p*-value, the two reported FDRs are compared, and *−* log(FDR) (base 10) is plotted against *−* log(cut-off *p*-value) (base 10).

Similar to PrSMs with one PTM, a mutated protein database was created to increase the number of identified PrSMs with two PTMs. Two glycine residues were added each protein in the ST protein database: one is at the one-third position of the protein; the other at the two-thirds position. MS-Align+ identified 2,404 complete PrSMs with two PTMs, and TD-GF was used to compute the spectral probabilities for the 2,404 PrSMs. The running time for computing spectral probabilities was 1,317 minutes (about 22 hours). Because it is extreme slow to find the best PrSM with two PTMs by searching a spectrum against a large random protein database with 10^6^ proteins, the evaluation method based on conditional spectral probabilities was not used. Only the evaluation method based on FDRs was applied. With all the 2,404 identified PrSMs, FDRs based on spectral probabilities and based on the target-decoy approach were computed for cut-off *p*-values in {0.0001, 0.0002, . . ., 1.0000}. When the cut-off *p*-value is smaller than 0.016 (*−* log *p*-value *>*1.80), the FDRs estimated by the two methods are similar. For 1% FDR, the target-decoy approach and the spectral probability approach estimated similar cut-off *p*-values 0.0164 and 0.0116, respectively. However, the FDRs based on spectral probabilities are not consistent with the "correct" FDRs (reported by the target-decoy approach) when the cut-off *p*-value is larger than 0.016 (Figure 3). One possible reason is that the filtering method implemented in MS-Align+ fails to keep the best PrSMs when their *p*-values are not small enough. From the above analysis, the spectral probabilities estimated by TD-GF are accurate when they are smaller than 0.016.

### Comparison with ProSightPC

All MS/MS spectra in the EC data set were deconvoluted by MS-Deconv [20]. The EC proteome database was downloaded from the Swiss-Prot database; a combined protein database was generated by concatenating the EC proteome database and a shuffled decoy database. To test the performance of TD-GF on proteoform identification, MS-Align+ coupled with TD-GF was applied to search the deconvoluted spectra against the combined database. The error tolerances for precursor masses and fragment masses were set as 15 ppm and two unknown PTMs were allowed. Using 1% spectrum-level FDR, 1,478 spectra were identified.

ProSightPC [10] was also applied to analyze the EC data set. ProSightPC provides several search modes for top-down spectral identification, including the absolute mass mode and the biomarker mode. Since some spectra in the EC data set were generated from truncated proteins, the biomarker mode was chosen for the analysis of the EC data set. The error tolerances for precursor masses and fragment masses were set as 15 ppm. ProSightPC identified 627 spectra with 1% spectrum-level FDR. All the 627 spectra were identified by MS-Align+ coupled with TD-GF. The test results show that MS-Align+ coupled with TD-GF outperformed the biomarker mode of ProSightPC.

## Conclusions

The experiments showed that the extended generating function method achieves high accuracy in computing spectral probabilities of PrSMs with PTMs. It is a non-trivial extension of the generating function method proposed in [8]. With accurate spectral probabilities and *E*-values, one can easily choose the correct PrSM from several candidate PrSMs for a spectrum, as well as separate correct PrSMs from incorrect ones identified from a large number of spectra. In addition, it provides a way to evaluate single PrSMs efficiently.
